# Satisfaction with Stroke Care Among Patients with Alzheimer’s and Other Dementias: A Swedish Register-Based Study

**DOI:** 10.3233/JAD-200976

**Published:** 2021-01-19

**Authors:** Minh Tuan Hoang, Ingemar Kåreholt, Mia von Euler, Lena von Koch, Maria Eriksdotter, Sara Garcia-Ptacek

**Affiliations:** a Division of Clinical Geriatrics, Department of Neurobiology, Care Sciences and Society, Karolinska Institutet, Stockholm, Sweden; b Aging Research Center (ARC), Karolinska Institutet and Stockholm University, Stockholm, Sweden; c Institute of Gerontology, School of Health and Welfare, Aging Research Network Jönköping (ARN-J), Jönköping University, Jönköping, Sweden; d Department of Medical Sciences, School of Medicine, Örebro University, Örebro, Sweden; e Division of Family Medicine and Primary Care, Department of Neurobiology, Care Sciences and Society, Karolinska Institutet, Stockholm, Sweden; f Neuro Theme, Karolinska University Hospital, Stockholm, Sweden; g Aging Theme, Karolinska University Hospital, Stockholm, Sweden; h Section for Neurology, Department of Internal Medicine, Södersjukhuset, Stockholm, Sweden

**Keywords:** Care, dementia, patient-reported, rehabilitation, satisfaction, stroke

## Abstract

**Background:**

Patient dissatisfaction with stroke care is associated with poor self-rated health and unmet care needs. Dementia patients’ satisfaction with stroke care is understudied.

**Objective:**

To compare satisfaction with stroke care in patients with and without dementia.

**Methods:**

This longitudinal cohort study included 5,932 dementia patients (2007–2017) who suffered a first stroke after dementia diagnosis and 39,457 non-dementia stroke patients (2007–2017). Data were retrieved by linking the Swedish Stroke Register, the Swedish Dementia Register, the Swedish National Patient Register, and the Swedish Prescribed Drug Register. The association between dementia and satisfaction was analyzed with ordinal logistic regression.

**Results:**

When dementia patients answered themselves, they reported significantly lower odds of satisfaction with acute stroke care (OR: 0.71; 95% CI: 0.60–0.85), healthcare staff’s attitude (OR: 0.79; 95% CI: 0.66–0.96), communication with doctors (OR: 0.78; 95% CI: 0.66–0.92), stroke information (OR: 0.62; 95% CI: 0.52–0.74); but not regarding inpatient rehabilitation (OR: 0.93; 95% CI: 0.75–1.16), or outpatient rehabilitation (OR: 0.93; 95% CI: 0.73–1.18). When patients answered with caregivers’ help, the association between dementia status and satisfaction remained significant in all items. Subgroup analyses showed that patients with Alzheimer’s disease and mixed dementia reported lower odds of satisfaction with acute care and healthcare staff’s attitude when they answered themselves.

**Conclusion:**

Patients with dementia reported lower satisfaction with stroke care, revealing unfulfilled care needs among dementia patients, which are possibly due to different (or less) care, or because dementia patients require adaptations to standard care.

## INTRODUCTION

Patient satisfaction with health care services is an integral indicator of health care quality [1, 2]. Lower patient satisfaction with stroke care was associated with older age, depressed mood, poor functioning, and poor self-rated health [3, 4]. It also related to emotional distress and unmet care needs [5, 6]. Better health outcomes and quality of life were associated with higher satisfaction with stroke care and rehabilitation [3, 6–9]. Previous studies on patient satisfaction six months after stroke showed that the highest rates of dissatisfaction were related to stroke care, rehabilitation, information, and services after discharge [10, 11].

Dementia, a frequent comorbidity of stroke, leads to lower functioning and survival after stroke [12–14]. Notwithstanding, dementia patients’ satisfaction with stroke care were neither included nor specifically mentioned in previous studies [3, 9]. Our study aimed to investigate the association of dementia with patient satisfaction with stroke care. This study belongs to a larger project which uses data from the Swedish Dementia and Stroke Registers to investigate all aspects of stroke care among patients with dementia in Sweden [12, 15–18].

## METHODS

### Study setting

In this longitudinal cohort study, data were ret-rieved by merging the Swedish Dementia Register(SveDem) and the Swedish Stroke Register (Riks-stroke) via patients’ identity number. The Swedish National Patient Register (NPR) and the Swedish Prescribed Drug Register (PDR) were used to find dementia patients who had escaped registration in SveDem.

Launched in 2007, SveDem is a Swedish national quality register for dementia, including data on dementia patients at the time of dementia diagnosis (made according to the International Classification of Diseases, Tenth Revision (ICD-10) codes) and annual follow-ups [19]. SveDem collects information about demographics, diagnosis, living situation, medication, and cognition levels [19]. Over 90,000 registrations and more than 57,000 follow-ups are recorded [20]. The coverage is estimated to be 30–35% of all dementia patients in Sweden based on an estimated dementia incidence in the different regions of Sweden [20, 21]. SveDem is the world’s largest dementia register.

Riksstroke is a national quality register for stroke care in Sweden [22, 23]. Adult patients diagnosed with intracerebral hemorrhage (ICD-10 I61), ischemic stroke (ICD-10 I63), or unspecified acute cerebrovascular event (ICD-10 I64) are registered in Riksstroke [22, 23]. Riksstroke includes data on each patient at the time of stroke, during acute care, and three-months and one-year follow-ups [22, 23]. Coverage rates of Riksstroke are over 90% at baseline registration, and 80–90% in three-months follow-ups [12, 22, 23]. With more than 450,000 events recorded until 2014, Riksstroke is one of the world’s largest stroke registers [22, 23].

### Study participants

**Fig. 1:**
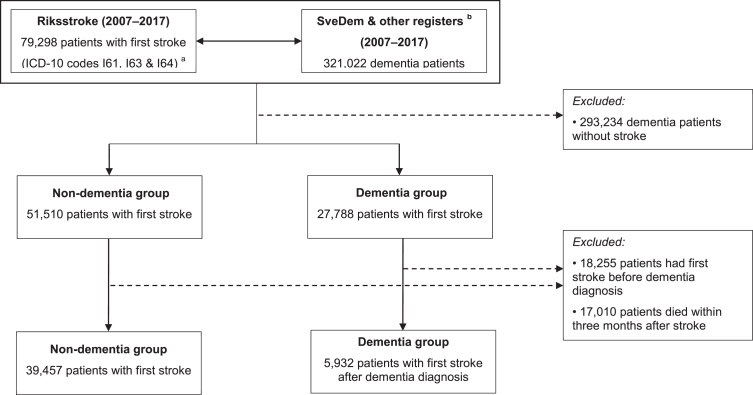
Patient selection process.^a^ International Classification of Diseases, Tenth Revision codes. I61: hemorrhage stroke. I63: ischemic stroke. I64: unspecified stroke.^b^ Dementia patients were identified from the Swedish Dementia Register (SveDem), the National Patient Register and the Swedish Prescribed Drug Register.

Data of 321,022 dementia patients identified in SveDem, the NPR, and the PDR (2007–2017) were linked with Riksstroke (2007–2017) to form two research groups. The dementia group encompassed patients who were registered with a dementia di-agnosis either in SveDem or the NPR, or who tookanti-dementia medications (Anatomical TherapeuticChemical Classification (ATC) System codes N06DX and N06DA) in the PDR. Only dementia pat-ients suffering a first stroke (ICD codes I61, I63, and I64) after the dementia diagnosis were included. Controls were defined as non-dementia patients who suffered a first stroke. Patients who suffered recurrent strokes or stroke before dementia diagnosis were excluded. Patient questionnaires were comple-ted three months after stroke onset and registered by Riksstroke. For this reason, patients who had died within three months after the stroke were ex-cluded. This left 5,932 patients in the dementiagroup and 39,457 non-dementia stroke controls for analysis (Fig. 1). Subgroup analyses were conducted in patients with Alzheimer’s disease and mixed dementia (Alzheimer’s and vascular). Because the information from the NPR and PDR is less reliable for specific diagnoses, only patients registered in SveDem were included in these subgroup analyses (961 patients with Alzheimer’s disease or mixed dementia versus 39,457 controls).

### Exposures

Dementia and stroke diagnoses, age at stroke, and sex were retrieved from SveDem, Riksstroke, NPR, and PDR. The Charlson Comorbidity Index (CCI) before stroke was calculated based on information from the NPR [24]. Consciousness at hospital admission, assessed through the Reaction Level Scale (RLS), is registered in Riksstroke at three levels: fully awake (RLS 1), drowsy (RLS 2-3), and unconscious (RLS 4–8) [22, 25]. Activities of daily living (ADL) before stroke (dressing, mobility, and toileting); and other features during acute care (complications, length of stay in acute care, places of discharge) were recorded from the Riksstroke hospital-reported acute care protocols [22].

Riksstroke also contributed three-months follow-up characteristics, which included ADL, self-rated health, difficulty with speaking, reading, and writing. The modified Rankin Scale (mRS) is a clinician-reported measure of disability which ranges from 0 to 6 [26, 27]. A higher mRS represents worse disability. The mRS was adapted from five Riksstroke questions on disability according to a validated Riksstroke conversion method [26]. People with mRS equal to 6, which represents death, were excluded.

### Outcomes

Outcomes included satisfaction with 1) acute st-roke care at hospital, 2) inpatient rehabilitation, 3) outpatient rehabilitation, 4) healthcare staff’s attitude, 5) communication with doctors, and 6) stroke information. These variables were extracted from Riksstroke three-months follow-up questionnaire, which was a questionnaire sent to patients’ homes or a telephone survey conducted by healthcare staff from the hospital where patients had been treated [22]. Patients could answer the survey completely by themselves or with help from caregivers (their family or healthcare staff). If patients were incapable of answering, their family or healthcare staff were asked to complete the questionnaire [22]. Possible answers to satisfaction questions were *very satisfied, satisfied, dissatisfied, very dissatisfied, had a need but did not receive this type of care, did not need* or *don*’*t know*. *Had a need but did not receive this type of care* was regarded as the lowest level of satisfaction. *Don*’*t know* was coded as missing.

### Statistical analyses

Means, standard deviation, and *t*-test were applied for numerical variables, unless otherwise specified. Categorical variables were presented as number of cases and percentages. Pearson’s Chi square or Fi-sher’s exact test were applied to examine the differences between two groups.

The association between patient satisfaction and dementia was investigated by ordinal logistic regressions and stratified by types of respondent (patient answering themselves, patient answering with the help of their caregivers, patient’s family, or healthcare staff). Unadjusted and partly adjusted models (adjusted for age at time of stroke and sex) were conducted as sensitivity analyses to examine the robustness of the results. The fully adjusted models were controlled for age at stroke, sex and pre-stroke CCI, attributes during acute care (consciousness at hospital admission, complications, length of stay in acute care, and places of discharge); characteristics three month after stroke (mRS, self-rated health, having difficulty with reading, speaking and writing). Subgroup analyses were performed to compare patients with Alzheimer’s disease or mixed dementia and non-dementia controls. Odds ratio (OR) and 95% confidence interval (95% CI) were reported.

All statistical tests were two tailed with a *p*-value <0.05 considered statistically significant. STATA version 15.1 (copyright StataCorp LLC, College Station, Texas, USA) was employed to perform the statistical analyses in this study. Missing data were handled by excluding cases listwise.

### Ethical considerations

Patients are informed about their inclusion in SveDem or Riksstroke and can decline registration or ask to be removed from the SveDem or Riksstroke at any time. This research project was approved by the Stockholm Ethics Committee (2015/743–31/4). Patient information was anonymized, and personal numbers were blinded to the researchers.

## RESULTS

### Patient characteristics before stroke, during acute stroke care, and three months after stroke

As shown in the Table 1, before stroke, dementia patients were more dependent in ADL than controls (*p* <  0.001): dressing without help (58.0% versus 93.0%), mobility without help (58.1% versus 91.4%), and toileting without help (64.7% versus 94.7%). Consciousness at hospital admission differed significantly: 82.7% of dementia patients were fully awake, compared with 90.6% of the non-dementia group.

**Table 1:** Patients’ characteristics before and during acute stroke care (*n* = 453,89)

Characteristics	Dementia	Non-Dementia	p^*^
	(*n* = 5,932)	(*n* = 39,457)
Age at stroke, mean (SD), y	82.1 (7.4)	81.1 (7.7)	<0.001
Sex, No. (%), women	3,468 (58.5)	21,576 (54.7)	<0.001
*Before stroke*
Charlson Comorbidity Index, median (IQR)	2 (2)	1 (2)	<0.001
Dressing, No. (%)
without help	3,441 (58.0)	36,702 (93.0)	<0.001
with help	2,135 (36.0)	2,284 (5.8)
missing	356 (6.0)	471 (1.2)
Mobility, No. (%)
without help in- &outdoors	3,448 (58.1)	36,067 (91.4)	<0.001
with help outdoors	1,480 (25.0)	2,160 (5.5)
with help	773 (13.0)	872 (2.2)
missing	231 (3.9)	358 (0.9)
Toileting, No. (%)
without help	3,837 (64.7)	37,360 (94.7)	<0.001
with help	1,750 (29.5)	1,656 (4.2)
missing	345 (5.8)	441 (1.1)
*During acute care*
Consciousness at hospital admission, No. (%)
fully awake	4,908 (82.7)	35,729 (90.6)	<0.001
drowsy	820 (13.8)	2,978 (7.6)
unconscious	122 (2.1)	410 (1.0)
missing	82 (1.4)	340 (0.8)
Complications, No. (%)	168 (2.8)	1,105 (2.8)	0.732
Discharge after acute care, No. (%)
home	2,024 (34.1)	25,636 (64.3)	<0.001
special accommodation	3,088 (52.1)	7,035 (17.8)
inpatient rehabilitation	720 (12.1)	6,104 (15.5)
other	95 (1.6)	911 (2.3)
missing	5 (0.1)	41 (0.1)
Length of stay in acute care, median (IQR), days	8 (10)	7 (10)	0.382

SD, standard deviation; IQR, interquartile range. ^*^ *p*-values were calculated with *t*-test (age at stroke), Mann-Whitney U test (Charlson Comorbidity Index and length of stay in acute care) and Pearson Chi-square test (the other variables).

Table 2 showed patients’ characteristics three months after stroke. Dementia patients were more disabled as shown by 69.4% of dementia patients having mRS scores >2, compared to 48.0% of non-dementia controls (*p* <  0.001). The proportion of patients who had difficulty with speaking, reading, or writing was significantly higher in the dementia group. Self-rated health was worse in the dementia group, compared to the non-dementia group (37.7% reported good health in the dementia group versus 64.3% of controls, *p* <  0.001).

**Table 2:** Patients’ functioning three months after stroke (*n* = 45,389)

Variable	Dementia	Non-Dementia	p^*^
	(*n* = 5,932)	(*n* = 39,457)
Dressing, No. (%)
without help	1,751 (29.5)	26,201 (66.4)	<0.001
with help	2,811 (47.4)	8,291 (21.0)
missing	1,370 (23.1)	4,965 (12.6)
Mobility, No. (%)
without help in- &outdoors	1,522 (25.7)	22,335 (56.6)	<0.001
with help only indoors	1,159 (19.5)	6,321 (16.0)
with help	1,879 (31.7)	5,798 (14.7)
missing	1,372 (23.1)	5,003 (12.7)
Toileting, No. (%)
without help	2,065 (34.8)	27,731 (70.3)	<0.001
with help	2,491 (42.0)	6,785 (17.2)
missing	1,376 (23.2)	4,941 (12.5)
modified Rankin Scale, No. (%)
0-1-2	493 (8.3)	15,660 (39.7)	<0.001
3	1,160 (19.6)	10,053 (25.5)
4	1,441 (24.3)	4,868 (12.3)
5	1,513 (25.5)	4,018 (10.2)
missing	1,325 (22.3)	4,858 (12.3)
Difficulty with speaking, No. (%)	1,316 (22.2)	5,578 (14.1)	<0.001
Difficulty with reading, No. (%)	1,512 (25.5)	6,224 (15.8)	<0.001
Difficulty with writing, No. (%)	1,958 (33.0)	8,219 (20.8)	<0.001
Self-rated health, No. (%)
very good	158 (2.7)	3,659 (9.3)	<0.001
pretty good	2,076 (35.0)	21,694 (55.0)
pretty bad	1,157 (19.5)	5,535 (14.0)
very bad	428 (7.2)	1,421 (3.6)
missing	1,613 (35.6)	7,148 (18.1)
Respondents, No. (%)
patients themselves	589 (9.9)	19,648 (49.8)	<0.001
patients with caregivers’ help	1,743 (29.4)	1,0811 (27.4)
patients’ family	1,479 (24.9)	3,081 (7.8)
healthcare staff	1,506 (25.4)	3,466 (8.8)
missing	615 (10.4)	2,451 (6.2)

^*^ *p*-values were calculated with Pearson Chi-square test.

### Difference in patient satisfaction with stroke care between dementia and non-dementia groups

The type of respondent to the questionnaire dif-fered significantly between dementia and non-dem-entia groups (Table 2). Fewer patients in the dementia group responded to the questionnaire by themselves: 9.9% among dementia patient versus 49.8% in non-dementia patients, 29.4% versus 27.4% answered with the help of caregivers, and 50.3% versus 16.6% answered by family or healthcare staff.

As shown in Table 3, when patients answered themselves, satisfaction in the dementia group was significantly lower than among non-dementia counterparts for acute care at hospital, inpatient rehabilitation, healthcare staff’s attitude, communication with doctors and stroke information. The proportion of either *very satisfied* or *satisfied* was: acute stroke care at hospital (87.6% versus 95.0%), inpatient rehabilitation (50.7% versus 53.1%), healthcare staff’s attitude (90.8% versus 96.3%), communication with doctors (62.3% versus 77.3%), stroke information (55.0% versus 73.2%). A greater proportion of the dementia group responding themselves stated that they *had a need but did not receive* a certain type of care: inpatient rehabilitation (3.2% versus 2.7%), outpatient rehabilitation (5.3% versus 4.1%), communication with doctors (17.3% versus 13.1%), and stroke information (23.1% versus 14.9%).

**Table 3:** Patient satisfaction with stroke care between the dementia (*n* = 5,932) and non-dementia groups (*n* = 39,457)

	Patients answered themselves		Patients answered with caregivers’ help		Patients’ family answered		Health care staff answered	
	*Dementia*	*Non-dementia*	*Dementia*	*Non-dementia*	*Dementia*	*Non-dementia*	*Dementia*	*Non-dementia*
	*(n* = *589)*	*(n* = *19,648)*	*(n* = *1,734)*	*(n* = *10,811)*	*(n* = *1,479)*	*(n* = *3,081)*	*(n* = *1,506)*	*(n* = *3,466)*
*Acute stroke care, No. (%)*
very satisfied	280 (47.5)	12,311 (62.7)	480 (27.5)	4,106 (38.0)	335 (22.7)	850 (27.6)	21 (1.4)	34 (1.0)
satisfied	236 (40.1)	6,356 (32.3)	900 (51.6)	5,459 (50.5)	730 (49.3)	1,417 (46.0)	60 (4.0)	92 (2.7)
dissatisfied	25 (4.2)	352 (1.8)	91 (5.2)	498 (4.6)	59 (4.0)	147 (4.8)	3 (0.2)	11 (0.3)
very dissatisfied	5 (0.9)	75 (0.4)	25 (1.5)	96 (0.9)	23 (1.6)	60 (1.9)	1 (0.0)	4 (0.1)
p^*^	<0.001		<0.001		0.004		0.546
missing	43 (7.3)	554 (2.8)	247 (14.2)	652 (6.0)	332 (22.4)	607 (19.7)	1,421 (94.4)	3,325 (95.9)
*Inpatient rehabilitation* ^†^*, No. (%)*
very satisfied	111 (18.9)	4,481 (22.8)	136 (7.8)	1,728 (16.0)	73 (4.9)	314 (10.2)	4 (0.2)	15 (0.4)
satisfied	187 (31.8)	5,954 (30.3)	552 (31.7)	4,322 (40.0)	385 (26.0)	984 (31.9)	42 (2.8)	69 (2.0)
dissatisfied	19 (3.2)	424 (2.2)	111 (6.4)	682 (6.3)	75 (5.1)	196 (6.4)	4 (0.3)	8 (0.2)
very dissatisfied	4 (0.7)	68 (0.3)	28 (1.6)	137 (1.2)	20 (1.4)	61 (2.0)	0 (0)	0 (0)
did not receive	19 (3.2)	521 (2.7)	136 (7.8)	690 (6.4)	115 (7.8)	201 (6.5)	11 (0.7)	9 (0.3)
p^*^	0.030		<0.001		<0.001		0.190
did not need	118 (20.0)	5,806 (29.6)	222 (12.7)	1,249 (11.6)	120 (8.1)	174 (5.7)	32 (2.1)	34 (0.1)
missing	131 (22.2)	2,394 (12.1)	558 (32.0)	2,003 (18.5)	691 (46.7)	1,151 (37.4)	1,413 (93.8)	3,331 (96.1)
*Outpatient rehabilitation* ^†^*, No. (%)*
very satisfied	76 (12.9)	2,778 (14.1)	118 (6.8)	1,182 (10.9)	47 (3.2)	188 (6.1)	10 (0.7)	13 (0.4)
satisfied	124 (21.0)	3,826 (19.5)	433 (24.8)	3,068 (28.4)	260 (17.6)	659 (21.4)	71 (4.7)	86 (0.5)
dissatisfied	18 (3.1)	512 (2.6)	153 (8.8)	765 (7.1)	115 (7.8)	275 (8.9)	2 (0.1)	11 (0.3)
very dissatisfied	7 (1.2)	105 (0.5)	43 (2.5)	245 (2.3)	42 (2.8)	111 (3.6)	2 (0.1)	3 (0.1)
did not receive	31 (5.3)	799 (4.1)	172 (9.9)	861 (8.0)	159 (10.8)	255 (8.3)	12 (0.8)	11 (0.3)
p^*^	0.137		<0.001		<0.001		0.264
did not need	168 (28.5)	7,525 (38.3)	278 (16.0)	1,977 (18.3)	154 (10.4)	291 (9.4)	64 (4.3)	64 (1.9)
missing	165 (28.0)	4,103 (20.9)	546 (31.2)	2,713 (25.0)	702 (47.5)	1,302 (42.3)	1,345 (89.3)	3,278 (94.6)
*Healthcare staff*’*s attitude, No. (%)*
very satisfied	354 (60.1)	1,4346 (73.0)	678 (38.9)	5,549 (51.3)	496 (33.5)	1,150 (37.3)	22 (1.5)	36 (1.0)
satisfied	181 (30.7)	4,569 (23.3)	755 (43.3)	4,343 (40.2)	588 (39.8)	1,296 (42.1)	56 (3.7)	89 (2.6)
dissatisfied	9 (1.5)	214 (1.1)	48 (2.8)	282 (2.6)	52 (3.5)	85 (2.8)	2 (0.1)	5 (0.2)
very dissatisfied	1 (0.2)	36 (0.2)	16 (0.9)	50 (0.5)	16 (1.1)	20 (0.7)	2 (0.1)	3 (0.1)
p^*^	<0.001		<0.001		0.081		0.987
missing	44 (7.5)	483 (2.4)	246 (14.1)	587 (5.4)	327 (22.1)	530 (17.1)	1,424 (94.6)	3,333 (96.1)
*Communication with doctors* ^†^*, No. (%)*
very satisfied	163 (27.7)	7,945 (40.4)	244 (14.0)	2,378 (22.0)	180 (12.2)	455 (14.8)	7 (0.5)	15 (0.4)
satisfied	204 (34.6)	7,258 (36.9)	614 (35.1)	4,305 (39.8)	404 (27.3)	953 (30.9)	29 (1.9)	57 (1.7)
dissatisfied	23 (3.9)	595 (3.0)	74 (4.3)	465 (4.3)	52 (3.5)	126 (4.1)	1 (0.1)	3 (0.1)
very dissatisfied	4 (0.7)	84 (0.5)	14 (0.8)	121 (1.1)	21 (1.4)	33 (1.1)	2 (0.1)	1 (0.0)
did not receive	102 (17.3)	2,569 (13.1)	292 (16.8)	1,906 (17.7)	227 (15.4)	485 (15.7)	18 (1.2)	25 (0.7)
p^*^	<0.001		<0.001		0.390		0.678
missing	93 (15.8)	1,197 (6.1)	505 (29.0)	1,636 (15.1)	595 (40.2)	1,029 (33.4)	1,449 (96.2)	3,365 (97.1)
*Stroke information* ^†^*, No. (%)*
very satisfied	88 (14.9)	5,150 (26.2)	130 (7.5)	1,234 (11.4)	97 (6.6)	295 (9.5)	5 (0.3)	8 (0.2)
satisfied	236 (40.1)	9,239 (47.0)	578 (33.2)	4,603 (42.6)	437 (29.6)	1,025 (33.3)	28 (1.9)	72 (2.1)
dissatisfied	24 (4.1)	773 (4.0)	118 (6.8)	813 (7.5)	58 (3.9)	170 (5.5)	2 (0.1)	5 (0.2)
very dissatisfied	4 (0.7)	81 (0.4)	26 (1.5)	150 (1.4)	18 (1.2)	46 (1.5)	2 (0.1)	2 (0.1)
did not receive	136 (23.1)	2,930 (14.9)	344 (19.7)	2,227 (20.6)	261 (17.6)	437 (14.2)	14 (1.0)	19 (0.5)
p^*^	<0.001		0.002		<0.001		0.471
missing	101 (17.1)	1,475 (7.5)	547 (31.3)	1,784 (16.5)	608 (41.1)	1,108 (36.0)	1,455 (96.6)	3,360 (96.9)

^*^ Pearson Chi-square test was used to compare the frequency of different levels of satisfaction between dementia and non-dementia groups. Fisher’s exact test was employed if satisfaction was answered by healthcare staff, because expected counts were less than 5.^†^ *Had a need but did not receive,* which is only available in satisfaction with inpatient, outpatient rehabilitation, conversation with doctors and stroke information, was considered as the lowest level of satisfaction.

When patients answered with caregivers’ help, a significantly lower proportion reported being either *very satisfied* or *satisfied* in the dementia group for all satisfaction items. The difference in satisfa-ction between the two groups was larger when the patients answered with help of caregivers than when they answered themselves. In terms of proxy answers, family-reported satisfaction was lower in the dementia group, whereas there were no significant differences among healthcare staff-reported satisfaction (Table 3).

### Association between patient satisfaction with stroke care and dementia

As summarized in Table 4, the dementia group was significantly less satisfied with the stroke care in the unadjusted, the age- and sex-adjusted, and the fully adjusted models (patients reported themselves and with help). In the fully adjusted models, dementia patients who answered themselves reported significantly lower odds of satisfaction with acute stroke care at hospital (adjusted OR: 0.71; 95% CI: 0.60–0.85, p <  0.001), healthcare staff’s attitude (adjusted OR: 0.79; 95% CI: 0.66–0.96, p = 0.015), communication with doctors (adjusted OR: 0.78; 95% CI: 0.66–0.92, p = 0.004), stroke information (adjusted OR: 0.62; 95% CI: 0.52–0.74, p <  0.001); but not regarding inpatient rehabilitation (adjusted OR: 0.93; 95% CI: 0.75–1.16, p = 0.529), or outpatient rehabilitation (adjusted OR: 0.93; 95% CI: 0.73 –1.18, p = 0.560).

**Table 4:** Association between dementia and patient satisfaction with stroke care (5,932 patients with dementia versus 39,457 non-dementia controls)

	Patients answered themselves	Patients answered with caregivers’ help	Patients’ family answered	Healthcare staff answered
Acute stroke care ^*^
Model 1	0.56 (0.47 –0.67) ^‡^	0.70 (0.63 –0.78) ^‡^	0.86 (0.75 –0.99)^§^	1.24 (0.71 –2.18)
Model 2	0.56 (0.47 –0.66) ^‡^	0.69 (0.62 –0.77) ^‡^	0.87 (0.75 –1.00)^§^	1.24 (0.70 –2.19)
Model 3	0.71 (0.60 –0.85) ^‡^	0.84 (0.75 –0.95)^§^	0.94 (0.80 –1.10)	1.67 (0.84 –3.33)
*Inpatient rehabilitation* ^†^
Model 1	0.74 (0.60 –0.91)^§^	0.60 (0.52 –0.68) ^‡^	0.68 (0.58 –0.81) ^‡^	0.53 (0.27 –1.04)
Model 2	0.72 (0.58 –0.89)^§^	0.57 (0.50 –0.65) ^‡^	0.68 (0.58 –0.81) ^‡^	0.54 (0.27 –1.07)
Model 3	0.93 (0.75 –1.16)	0.76 (0.66 –0.87) ^‡^	0.86 (0.71 –1.04)	0.77 (0.31 –1.88)
*Outpatient rehabilitation* ^†^
Model 1	0.78 (0.62 –0.99)^§^	0.66 (0.58 –0.75) ^‡^	0.68 (0.57 –0.80) ^‡^	1.10 (0.62 –1.96)
Model 2	0.76 (0.60 –0.96)^§^	0.62 (0.55 –0.71) ^‡^	0.67 (0.56 –0.79) ^‡^	1.08 (0.61 –1.94)
Model 3	0.93 (0.73 –1.18)	0.73 (0.64 –0.84) ^‡^	0.71 (0.59 –0.86) ^‡^	1.13 (0.56 –2.26)
*Healthcare*
staff’s attitude ^*^
Model 1	0.62 (0.52 –0.75) ^‡^	0.70 (0.63 –0.78) ^‡^	0.89 (0.78 –1.02)	1.03 (0.57 –1.83)
Model 2	0.62 (0.52 –0.74) ^‡^	0.69 (0.62 –0.77) ^‡^	0.89 (0.78 –1.03)	1.06 (0.59 –1.92)
Model 3	0.79 (0.66 –0.96)^§^	0.84 (0.75 –0.94)^§^	0.99 (0.85 –1.16)	1.36 (0.65 –2.84)
*Communication *
*with doctors*^†^
Model 1	0.63 (0.54 –0.75) ^‡^	0.78 (0.70 –0.88) ^‡^	0.89 (0.77 –1.03)	0.72 (0.39 –1.35)
Model 2	0.62 (0.53 –0.74) ^‡^	0.76 (0.68 –0.85) ^‡^	0.88 (0.76 –1.02)	0.70 (0.37 –1.31)
Model 3	0.78 (0.66 –0.92)^§^	0.87 (0.77 –0.98)^§^	0.96 (0.82 –1.13)	0.52 (0.23 –1.15)
*Stroke information* ^†^
Model 1	0.53 (0.44 –0.62) ^‡^	0.79 (0.71 –0.89) ^‡^	0.73 (0.63 –0.85) ^‡^	0.69 (0.35 –1.37)
Model 2	0.51 (0.43 –0.60) ^‡^	0.74 (0.66 –0.83) ^‡^	0.73 (0.62 –0.84) ^‡^	0.73 (0.37 –1.45)
Model 3	0.62 (0.52 –0.74) ^‡^	0.89 (0.79 –1.00)^§^	0.85 (0.72 –1.00)	0.72 (0.31 –1.66)

Data are presented as odds ratios (95% confidence intervals), which represent levels of satisfaction among dementia patients compared to non-dementia patients. Model 1: Unadjusted ordinal logistic regression model. Model 2: Multivariable ordinal logistic regression model (adjusted by age at stroke and sex). Model 3: Multivariable ordinal logistic regression model (adjusted by age at stroke, sex, and pre-stroke characteristic: Charlson Comorbidity Index; features during acute care: consciousness at hospital admission, complications, length of stay in acute care, places of discharge after acute care. characteristics three month after stroke: modified Rankin Scale, self-rated health and having difficulty with reading, speaking, writing). ^*^Patients satisfaction levels included *very dissatisfied, dissatisfied, satisfied, and very satisfied.* ^†^Patient satisfaction levels encompassed *had a need but did not receive, very dissatisfied, dissatisfied, satisfied, and very satisfied*. ^‡^*p* < 0.001. ^§^*p* < 0.05.

When patients answered with the help of caregivers, the association between dementia status and satisfaction remained significant in all items. As for proxy-reported satisfaction, significant associations did not exist, except for satisfaction with outpatient rehabilitation (reported by patients’ family).

### Sensitivity and subgroup analyses

Supplementary Table 1 showed the comparison between patients with Alzheimer’s disease or mixed dementia and non-dementia counterparts. When patients answered themselves, there was a significant association between Alzheimer’s disease or mixed dementia and satisfaction with acute stroke care adjusted OR: 0.65; 95% CI: 0.42–0.99, *p* = 0.045) and healthcare staff’s attitude; adjusted OR: 0.60; 95% CI: 0.39–0.94, *p* = 0.025). When patients answered with caregivers’ help, patients with Alzheimer’s disease or mixed dementia reported significantly lowered odds of satisfaction with acute stroke care, inpatient rehabilitation, outpatient rehabilitation, and healthcare staff’s attitude, compared to controls. No significant association between satisfaction and Alzheimer’s disease or mixed dementia were found among proxy answers, except for healthcare staff-reported satisfaction with outpatient rehabilitation.

## DISCUSSION

This study examined the satisfaction of Alz-heimer’s disease and other dementias patients with stroke care, in comparison with non-dementia controls. When patients answered themselves or with caregivers’ help, dementia was significantly associated with lower satisfaction with stroke care. A lower proportion of dementia patients reported satisfactionwith acute stroke care, inpatient rehabilitation, outpatient rehabilitation, healthcare staff’s attitude, com-munication with doctors, and stroke information, compared to non-dementia controls. The proportions of dementia patients who were satisfied with stroke care were also lower compared to general stroke pa-tients in previous studies [3, 4] or in the Riksstroke annual reports (where 95.5% report being satisfied with care at hospital, 91.1% satisfied with inpatient rehabilitation, and 85.4% satisfied with outpatient rehabilitation) [28]. There is no consensus on the definition of patient satisfaction and no theory explaining the meaning of satisfaction [1, 2, 29, 30]. Satisfaction is, by its nature, subjective. It is influenced by a patient’s expectations and experience with the care which they receive. In cognitively impaired patients, the link between satisfaction and quality of provided care might be more tenuous, but it is still important to elicit information on patient satisfaction in order to improve care and make it more patient-centered. Furthermore, patient satisfaction not only mirrored real differences in the healthcare supply [4], but also correlated with patients’ perception of trust in healthcare professionals [31]. Thus, lower satisfaction with stroke care among dementia patients should be interpreted with caution.

First, unfulfilled care needs probably made dementia patients feel more dissatisfied than non-dementia patients. This explanation is plausible because some recent studies indicated that unmet health care needs were significantly associated with lower patientsatisfaction [5, 6]. Riksstroke also mentions in their annual report that not receiving any care or support after hospital discharge leads to patient dissatisfaction [28]. In our study, higher proportions of patients in dementia group reported that they *had a need but did not receive* inpatient rehabilitation, outpatient rehabilitation, communication with doctors, and stroke information compared with their counterparts. Thus, dementia patients might not receive the care, disease information, or rehabilitation that they need. From a clinical perspective, healthcare staff might assume less potential for recovery in dementia patients and provide less care and rehabilitation resources [32]. From the patient’s viewpoint, dementia patients might have different care needs. They may need different, simpler, or more frequent explanations on their condition and care plan. Reminders of care, disease information, or rehabilitation may also be more necessary for dementia patients. To a certain extent, increasing reminders and repetition of information may help in some cases, but probably not in advanced dementia.

Second, differences in the organization and delivery of stroke care with non-dementia patients might lead to lower patient satisfaction in the dementia group. The provision and organization of stroke services influenced patient satisfaction [3]. In addition, the difference in stroke care between dementia and non-dementia patients in Sweden was mentioned in recent studies from our group: dementia patients areless likely to receive some types of diagnostic assessments and have shorter lengths of stay in stroke unit and rehabilitation (which translated into lower expenditure for inpatient rehabilitation) [15, 16, 32, 33]. Access to thrombolysis depends more on mobility and functioning than dementia status in Sweden, although younger independent patients with dementia are less likely to receive thrombolysis [33]. These findings emphasize the importance of quality management on stroke care. Patient satisfaction is a legiti-mate indicator for improving the health care services,but also one of the strategic goals of health care system [34]. The lower satisfaction with stroke care in dementia patients possibly reflected a gap in the organization and delivery of stroke care. Further stu-dies on the provision and process of stroke care fordementia patients should be conducted; particularly qualitative studies to determine which factors increase patient satisfaction in cognitively impaired patients.

Third, lower satisfaction with stroke care among dementia patients might be explained by worse functioning. Three months after stroke, they were more dependent in ADL, more disabled (assessed by mRS scores), and had worse self-reported health. Moreover, difficulty with speaking, reading, and writing were more common in the dementia patients than their counterparts. Since we controlled for all these factors, it is probable that the difference between dementia and non-dementia patients depended on additional factors. Worse memory and cognitive impairment may have caused recall bias and dementia patients may not remember or recognize rehabilitation that they received. The meaning of the concept rehabilitation or what rehabilitation can entail may not be clear for all patients. Furthermore, low satisfaction with information received in health care is recurrently reported by authorities based on surveys in the general population [35]. This might influence satisfaction with rehabilitation, communication with doctors, or stroke information among patients who answered the questions themselves.

Finally, lower satisfaction with stroke care in the dementia group is probably due to higher proportions of patients who answered with the help of caregivers. Validation studies of Riksstroke showed that when caregivers helped patients answer questionnaires, patients appeared less satisfied compared to patients answering themselves, particularly during the first months after stroke [36, 37]. This fact was also confirmed with either dementia or stroke patients in other studies [38, 39]. In our study, lower proportion of dementia patients answered themselves, compared to non-dementia controls: 9.9% versus 49.8% patients answered alone, and 29.4% versus 27.4% patients answered with the help of caregivers. When patients responded to the questionnaire with the help of caregivers, they were significantly less satisfied than when patients responded themselves, in both dementia and non-dementia groups.

### Strengths and limitations

A study based on patient-reported outcomes faces several limitations. First, recall bias is particularly problematic in this study on patients with dementia. After a stroke, both patients with and without previous dementia may be cognitively and physically impaired. It might be a challenge for them to self-report their health and satisfaction and to distinguish between the different services in the stroke care trajectory, especially when the survey was carried out three months after stroke. However, patient satisfaction is an important part in increasing patient participation in care, including in patients with dementia, where participation is particularly threatened [40]. Accepting caregivers-supported responses is a way to increase both caregivers and patient participation [40]. Specific systematic errors possibly occurred in the study, particularly due to the evident differences in cognitive impairment between the dementia and non-dementia groups. Results obtained from dementia patients might not be fully comparable with results from the non-dementia group because the proportion of dementia patients completing the questionnaire by themselves was much lower among the dementia patients. Certain limitations of a register-based study cannot be eluded, such as missing values, incomplete data, etc. For instance, stroke severity (measured with the National Institutes of Health Stroke Scale NIHSS) was not considered because of incomplete Riksstroke data. This might distort the results of the study to some extent because stroke severity influences patient satisfaction. This weakness was addressed by adding consciousness at hospital admission as a covariate. Despite drawbacks, the linkage of large national databases with high coverage is the most notable strength of this study. A large, national cohort of dementia and stroke patients, with their self-reported perspectives collected from most of hospitals and health care centers in Sweden is an additional strength.

## Conclusions

Satisfaction with acute stroke care, inpatient rehabilitation, outpatient rehabilitation, healthcare staff’s attitude, communication with doctors, and stroke information was significantly lower among dementia patients compared to non-dementia controls.

Lower satisfaction with care may reflect unmet needs in the dementia population, possibly due to different (or less) care, or because dementia patients require adaptations to standard care. Policymakers and healthcare staff should examine and adapt the process of stroke care, rehabilitation, and clinician-patient communication to improve dementia patients’ satisfaction and health outcomes. There is a need for future research on the provision of stroke care and rehabilitation specifically for dementia patients.

## Supplementary Material

Supplementary Material
