# Supplementary material for: Satisfaction with Stroke Care Among Patients with Alzheimer’s and Other Dementias: A Swedish Register-Based Study
Source: J Alzheimers Dis. 2021 Jan 19;79(2):905–16. doi: 10.3233/JAD-200976 (PMC7902943; doi:10.3233/JAD-200976)
Supplement: Supplementary Material [file jad-79-jad200976-s001.pdf]

Supplementary Material

Satisfaction with Stroke Care Among Patients with Alzheimer's and Other Dementias: A Swedish Register-Based Study

Supplementary Table 1
Alzheimer's disease and mixed dementia in relation to patient satisfaction with stroke care (961 patients with Alzheimer's disease or mixed dementia versus 39,457 non-dementia controls)

	**Patients answered themselves**	**Patients answered with caregivers' help**	**Patients' family answered**	**Healthcare staff answered**
Acute stroke care*				
Model 1	0.67 (0.45 – 1.02) §	0.69 (0.54 – 0.89) §	0.82 (0.60 – 1.10)	1.69 (0.48 – 5.88)
Model 2	0.67 (0.44 – 1.01) §	0.68 (0.53 – 0.87) §	0.83 (0.61 – 1.12)	1.85 (0.50 – 6.86)
Model 3	0.65 (0.42 – 0.99) §	0.75 (0.58 – 0.97) §	0.93 (0.68 – 1.27)	2.01 (0.41 – 9.74)
Inpatient rehabilitation†				
Model 1	0.58 (0.34 – 0.98) §	0.45 (0.33 – 0.61) ‡	0.59 (0.40 – 0.87) §	0.41 (0.09 – 1.81)
Model 2	0.57 (0.33 – 0.96) §	0.43 (0.32 – 0.58) ‡	0.60 (0.41 – 0.88) §	0.43 (0.09 – 2.00)
Model 3	0.59 (0.34 – 1.01)	0.55 (0.41 – 0.75) ‡	0.77 (0.51 – 1.16)	0.89 (0.12 – 6.54)
Outpatient rehabilitation†				
Model 1	0.63 (0.36 – 1.10)	0.56 (0.42 – 0.75) ‡	0.69 (0.47 – 0.99) §	0.15 (0.03 – 0.64) §
Model 2	0.61 (0.35 – 1.08)	0.53 (0.39 – 0.70) ‡	0.68 (0.47 – 0.98) §	0.15 (0.03 – 0.69) §
Model 3	0.61 (0.34 – 1.08)	0.60 (0.44 – 0.80) ‡	0.71 (0.48 – 1.04)	0.13 (0.02 – 0.83) §
Healthcare staff's attitude*				
Model 1	0.59 (0.39 – 0.90) §	0.66 (0.52 – 0.85) §	0.77 (0.57 – 1.04)	1.95 (0.55 – 6.94)
Model 2	0.58 (0.38 – 0.89) §	0.65 (0.51 – 0.84) §	0.77 (0.57 – 1.04)	2.16 (0.58 – 8.14)
Model 3	0.60 (0.39 – 0.94) §	0.72 (0.56 – 0.93) §	0.87 (0.63 – 1.19)	2.02 (0.40 – 10.11)
Communication with doctors†				
Model 1	0.74 (0.50 – 1.10)	0.95 (0.74 – 1.22)	1.14 (0.81 – 1.60)	2.85 (0.45 – 17.88)
Model 2	0.72 (0.49 – 1.07)	0.92 (0.72 – 1.19)	1.11 (0.79 – 1.56)	1.79 (0.29 – 11.05)
Model 3	0.78 (0.52 – 1.16)	0.94 (0.73 – 1.22)	1.19 (0.85 – 1.69)	0.77 (0.10 – 5.79)
Stroke information†2				
Model 1	0.72 (0.48 – 1.09)	0.84 (0.65 – 1.08)	0.81 (0.58 – 1.14)	1.02 (0.17 – 6.16)
Model 2	0.69 (0.46 – 1.04)	0.78 (0.60 – 1.00)	0.79 (0.57 – 1.11)	1.33 (0.24 – 7.52)
Model 3	0.72 (0.47 – 1.08)	0.86 (0.66 – 1.12)	0.93 (0.66 – 1.32)	2.43 (0.34 – 17.23)

Data are presented as odds ratios (95% confidence intervals), which represent levels of satisfaction among dementia patients compared to non-dementia patients.
Model 1: Unadjusted ordinal logistic regression model
Model 2: Multivariable ordinal logistic regression model (adjusted by age at stroke and sex)
Model 3: Multivariable ordinal logistic regression model (adjusted by age at stroke, sex, and pre-stroke characteristic: Charlson Comorbidity Index; features during acute care: consciousness at hospital admission, complications, length of stay in acute care, discharge places after acute care; characteristics three month after stroke: modified Rankin Scale, self-rated health and having difficulty with reading, speaking, writing)
*Patients satisfaction levels included *very dissatisfied, dissatisfied, satisfied, and very satisfied.*
†Patient satisfaction levels encompassed *had a need but did not receive, very dissatisfied, dissatisfied, satisfied, and very satisfied*.
‡$p < 0.001$
§$p < 0.05$
